# ICD-11 Morbidity Pilot in Kuwait: Methodology and Lessons Learned for Future Implementation

**DOI:** 10.3390/ijerph19053057

**Published:** 2022-03-05

**Authors:** Islam Ibrahim, Mohammad Alrashidi, Mustafa Al-Salamin, Nenad Kostanjsek, Robert Jakob, Suhair Azam, Naela Al-Mazeedi, Fatima Al-Asoomi

**Affiliations:** 1 National Center for Health Information, Ministry of Health, Sulibekhat 13001, Kuwait; malsalameen@yahoo.com (M.A.-S.); suhairazam@gmail.com (S.A.); dralasoomi@yahoo.com (F.A.-A.); 2 Farwaniya Hospital, Ministry of Health, Farwaniya 81004, Kuwait; mralrashidi@moh.gov.kw (M.A.); naela@doctor.com (N.A.-M.); 3 World Health Organization, 1211 Geneva, Switzerland; kostanjsekn@who.int (N.K.); jakobr@who.int (R.J.)

**Keywords:** ICD-11, physician, morbidity coding, inpatient, pilot, Kuwait, training, electronic health information system

## Abstract

This paper reports on the first ICD-11 morbidity pilot for inpatient discharges in a public general hospital. We detail the pilot methodology, lessons learned in terms of facilitators and challenges, physician-reported opinion, and considerations for future implementation. The pilot included: engaging stakeholders; selecting the setting; building a common understanding of the discharge process; evaluating and preparing IT infrastructure; ICD-11 training; small-scale pre-pilot testing; implementing the pilot while providing on-site support and collecting data for analysis including a brief user-experience survey. Overall, physicians were satisfied with the experience. Facilitators for success included national health system influence, leadership commitment, a multidisciplinary team approach, physician-tailored training, using social media for training, and providing on-site support. Challenges included potential IT problems, and difficulties relating to training and engaging physicians. Issues to consider include DRG system requirements, and comparability of ICD-11 pilot results from different countries. In conclusion, ICD-11 can be successfully implemented for documenting diagnoses by physicians in a public hospital by installing the coding tool on the electronic hospital information system. Pilots can improve ICD-11 content by using the online proposal mechanism. Implementing ICD-11 requires effective change management, stakeholder-tailored communication, and innovative ideas for training to match the electronic nature of ICD-11 and its potential new users, physicians.

## 1. Introduction

The International Statistical Classification of Diseases and Related Health Problems (ICD) standardizes the way we report diagnoses and health conditions, enabling us to count, analyze, and present health data, producing health statistics for decision making at national and international levels [1]. Since the release of ICD-10 in the early 1990s, computers have become more affordable and the internet widely accessible, bringing digital innovation to the heart of every aspect of healthcare services [2]. This has dramatically changed the way health data is managed, creating new opportunities and requirements for different health systems. Electronic Health Information Systems (EHIS) and Electronic Health Information Exchange (HIE) mean we need an electronic ICD that provides an international standard to achieve semantic interoperability [3]. The wide range of country-specific ICD-10 modifications and medical terminologies means we need a classification that includes a wealth of synonyms and details to fulfill the needs of different countries to ensure internationally comparable morbidity data [4]. The growing reliance on casemix/Diagnosis Related Group (DRG) systems for reimbursement, benchmarking, and health service planning means we need a comprehensive package of WHO Family of Health Classifications (WHO-FIC) that reflects up-to-date clinical conditions and interventions in the field [5,6]. ICD-11, adopted by the World Health Assembly in May 2019, fulfills all that and more. As a global public good, it is also freely available to use and is regularly updated and maintained through a transparent mechanism in which experts and users from all over the world can take part [3].

The Ministry of Health (MOH) in Kuwait is the main funder, provider, and only regulator of healthcare for a population of 4.8 million [7,8]. ICD-10 (the international WHO version) is used for coding discharge diagnoses on standardized paper forms (abstracts of the discharge summary forms) delivered to the National Center for Health Information (NCHI). Data entry, analysis, and reporting are carried out at NCHI and are the basis of national morbidity health statistics. Kuwait was one of the early adopters of ICD-10, making the transition from ICD-9 in 1996. The MOH does not use a DRG system. The funding of healthcare services relies on annual budgets. Hence, morbidity coding is used for statistical purposes and not for provider reimbursement.

Countries with ICD legacy have traditionally started the transition process with a focus on dual coding, content coverage, and quality of coded data studies [9,10,11]. This traditional approach focuses on piloting the content of ICD-11 using the same methods used for ICD-10 coding, for example, by restricting the pilot to coding diagnoses in the medical record department by professional coders. However, ICD-11 is a major breakthrough compared to its predecessors. ICD-11 is completely electronic, accessible online or offline, and smoothly integrated into EHIS. With more than 130,000 medical terms, ICD-11 can be easily used by clinicians through a user-friendly interface, and is maintained through a transparent online proposal platform that allows users and experts from all over the world to directly contribute to improving its content [1]. The groundbreaking nature of ICD-11 calls for a novel approach to piloting and transitioning that involves hands-on implementation in real-life clinical settings where the ICD-11 coding tool is integrated into the EHIS, with clinician involvement, and direct feedback to improve ICD-11 content through the WHO online proposal mechanism. Reporting on ICD-11 pilots using the new approach should not be restricted to findings relating to ICD-11 content. Pilots also need to report on workflow changes and how different stakeholders interact with these new changes. This study aims to share the lessons learned in terms of facilitators and challenges from piloting the real-life implementation of ICD-11 for morbidity in a public general hospital in Kuwait so that other health systems, especially those that are similar to the one in Kuwait, can benefit as they start their own ICD-11 journeys.

## 2. Materials and Methods

The pilot was organized and conducted by the National Center for Health Information (NCHI), Ministry of Health (MOH), Kuwait. The center is the national body responsible for managing health data and providing statistical information for decision making at the national level. This includes setting standards for reporting health data. The center also serves as the WHO Collaborating Center for the WHO Family of International Classifications (WHO-FIC CC) in the WHO Eastern Mediterranean region. As such, NCHI is the National Centre of Excellence for ICD-11 implementation in Kuwait, providing focused leadership and collaborating efforts by all involved stakeholders for piloting ICD-11, as well as planning and implementing the transition from ICD-10 to ICD-11 in the country.

A core team of NCHI staff members was formed to manage the ICD-11 pilot project. The team’s first task was selecting the pilot setting. We selected Farwaniya hospital, which is a secondary public hospital funded and regulated by MOH. The hospital serves Farwaniya governorate, the most populous of Kuwait’s six governorates, with a population of 1,260,000, representing 26.3% of the country’s 2020 mid-year population. With 848 beds, the hospital served 27,684 inpatients in 2020 (the highest of all Kuwait’s hospitals), with an average length of stay of 6.1 days [8]. The hospital has an Electronic Health Information System (EHIS) into which the ICD-11 Application Programming Interface (ICD-11 API) and embedded ICD-11 Coding Tool were easily integrated [12]. In order to work on a manageable scale, it was decided that the pilot was to be restricted to inpatient services in the internal medicine department. The bed occupancy rate for the internal medicine department at the hospital (with 228 beds) was 74.9% in 2020, the maximum reported when compared to the internal medicine departments of all the other hospitals in the country [8].

Throughout the pilot, the existing system for national reporting of clinical diagnoses using ICD-10 was maintained to ensure that the stability of reporting remained unaffected. The existing system is based on a paper-form that functions as an abstract of the patient discharge summary in which the diagnosis documented by the treating physician at discharge is printed onto a paper form, then coded using ICD-10 by medical record clerks, and finally delivered to NCHI for data entry, statistical analysis, and reporting.

An initial meeting was held at the hospital between all key stakeholders: the NCHI core team, the hospital director, the heads of medical departments, representatives from the central IT directorate at MOH, and the hospital IT department. The aim of the meeting was to establish stakeholder engagement by promoting the benefits of transitioning to ICD-11 to all parties involved. To clinicians, the key message delivered by NCHI was “ICD-11 is scientifically up-to-date. It was made with the contribution of expert clinicians from all over the world. It uses user-friendly Google-like search with a wealth of synonyms and abbreviations making it more convenient than existing ICD-10 drop-down lists on the EHIS” [1]. To the IT professionals, the key message was “ICD-11 is fully electronic, ensuring interoperability with existing IT infrastructure” [1]. To the hospital administration, the key message was “ICD-11 will allow quicker statistical analysis and reporting because it is electronic and avoids the slow and labor-intensive paper-based current process. ICD-11 will overcome the near impossible task of using free text for producing reliable statistics on diagnoses and causes of injury for decision making.” An agreement was reached, and a multidisciplinary team was formed to carry out the pilot which included representatives from NCHI, hospital administration, hospital IT, and medical staff from the internal medicine department.

Before the pilot, it was necessary for the NCHI team to understand the inpatient discharge process from the point of view of the physician by shadowing them as they discharged inpatients from the internal medicine ward. All relevant EHIS fields on the discharge user interface (UI) were studied to determine what data elements were available and which ones were actually filled in by physicians. This guaranteed a common ground of understanding between all parties involved in the pilot.

The pilot was conducted over three main phases: preparation, implementation, and finally analysis and reporting.

### 2.1. The Preparation Phase

#### 2.1.1. Information and Technology Infrastructure

During the preparation phase, a series of meetings were held by the multidisciplinary pilot team to assess IT infrastructure needs. Computers were available at computer stations in the inpatient wards where physicians carry out their documentation at discharge. Due to data security concerns, the central IT department at MOH does not permit MOH computers with EHIS to connect to the internet, so the IT department decided to utilize Docker container technology in order to use the ICD-11 API offline [12].

The EHIS discharge diagnosis screen had free text fields for documenting diagnoses with no clear distinction between principal diagnosis and other/secondary diagnoses, a concept that was not part of the documentation culture in the hospital. The screen also included non-mandatory fields to select ICD-10 codes from a drop-down list based on ICD-10 volume 1 (Tabular List). The ICD-10 list was rarely used, as physicians complained that they often did not find the terms they were looking for. The team designed a new screen to improve the documentation of final diagnoses at discharge. The new screen made a clear distinction between the principal diagnosis (the top diagnosis field) and other diagnoses (all other diagnosis fields). For each diagnosis, two fields are filled: one for free texting the diagnosis, and one for the selected corresponding ICD-11 entity.

To be able to install the ICD API on the EHIS, the hospital IT staff received a short training session by IT professionals from MOH who had previously attended a webinar on the technical infrastructure of ICD-11 by the WHO Classifications and Terminologies Unit [13]. Participants were trained to install the ICD-API onto the hospital EHIS using the Docker container to enable it to work offline. After the training, the hospital IT created the new diagnosis screen based on the new requirements, and the ICD-11 coding tool successfully worked on the EHIS. It was then tested and approved by the NCHI team.

**Figure 1:**
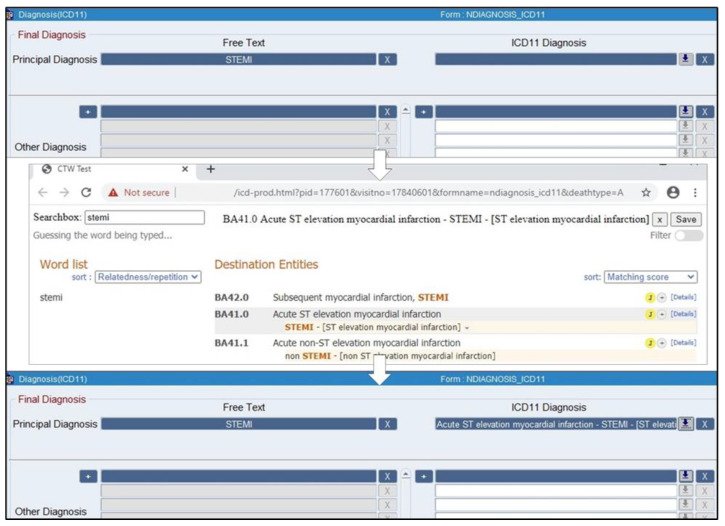
The new diagnosis screen on the EHIS.

During the pilot, the documentation of each diagnosis on the new screen involved the following steps: first the physician free texts the diagnosis in the free text field, then clicks an icon beside the corresponding ICD-11 field. This opens up a new window with another search box into which the physician types in the search terms (they can copy and paste the free text diagnosis). After selecting the correct ICD-11 entity, they click a save icon on the screen, and close this window, returning them to the diagnosis screen. The selected ICD-11 entity title will now automatically appear in the ICD-11 field on the diagnosis screen. The ICD-11 code and the corresponding Unique Resource Identifier (URI) are automatically saved in the database and will not appear on the final diagnosis screen. Figure 1 shows the new diagnosis screen on the EHIS. A demonstration of how to use it is included in the training video uploaded to the NCHI YouTube channel from 3:20 to 4:07 [14].

#### 2.1.2. ICD-11 Training

The following step was to train the physicians on ICD-11. The main request by the head of the internal medicine department was to keep the training short and simple, as physicians were overwhelmed and exhausted because of the COVID-19 pandemic. A two-hour training workshop was prepared by NCHI. This included a lecture followed by a hands-on practical exercise in a computer lab on the hospital EHIS. The workshop covered the following topics: the rationale for using disease classifications such as ICD-11, the physician’s role in diagnosis documentation, how to use the new EHIS diagnosis screen, the definition of the principal and other diagnoses with practical examples, the advantages of ICD-11 over ICD-10, and how to use ICD-11 for documenting diagnoses. Due to the short duration of the training, we focused on using the ICD-11 coding tool and its main features, namely using the word list to narrow down search results, adding details to a diagnosis using postcoordination, using abbreviations, and finally we showcased the fact that ICD-11 can automatically postcoordinate detailed diagnoses on its own. The training material used the hospital EHIS screens to ensure familiarity. The focus throughout the training was not on the codes; rather, it was on the fact that ICD-11 uses a Google-style search that enables physicians to standardize the way diagnoses are documented worldwide.

The target trainees were physicians responsible for discharging inpatients from the wards of the internal medicine department at the hospital. Training was conducted by a public health specialist physician from NCHI who was WHO ICD-11 ToT-trained, with prior experience in ICD coding and post-graduate teaching. The trainer’s medical background and familiarity with the quality of diagnosis documentation in different hospitals in the country ensured that they spoke a common language with the trainees.

Two training workshops were held in March 2021. Due to COVID-19 social distancing restrictions, the maximum number of participants allowed per workshop was 30 physicians. To allow more physicians to access the training, we recorded the training material in two short videos (around 10 min each): ICD-11 for physicians [14], and documentation of final diagnoses at discharge [15]. The videos were then disseminated via WhatsApp chat groups, as that is the most popular medium used by physicians for communication within each department in the hospital. As NCHI also functions as a WHO-FIC CC, we created a YouTube channel to share the training material with a wider international audience [16].

### 2.2. The Implementation Phase

Once the EHIS was ready with the new diagnosis screen (including the ICD-11 coding tool) and the physicians were trained to use it, we were ready to start the pilot.

#### 2.2.1. Small-Scale Pilot

For a period of one week (25–31 March), physicians had the choice between the old diagnosis screen and the new one. This period aimed to ensure that any IT issues were resolved and that physicians gradually became familiar with the new screen. Initially, the Docker container was on a separate computer not on the hospital server. This stopped working several times, so the container was moved to the main server. From 1 April onwards, after all IT issues were resolved, the old diagnosis screen was inactivated, and only the new discharge screen with ICD-11 was available for use at the wards where the pilot took place.

#### 2.2.2. On-Site Support

A team was assigned to provide on-site support during implementation. The team was formed of NCHI staff, including two physicians to provide ICD-11 support, and one IT staff member from the hospital IT department. During the small scale-pilot and the first week of implementation (25 March–8 April), meetings were held at the end of each day between the support team, the hospital director, and the head of WHO-FIC CC to discuss: problems encountered during the day, ways to solve them, and the plan for the following day. Following this period, meetings were held twice a week, and from the end of April onwards until the end of the pilot, they were only held when needed. Until the end of May, both NCHI physicians conducted at least two daily rounds on all computer workstations in the pilot wards. The aim was to answer questions from physicians as they used ICD-11, and to provide one-to-one training for those who did not attend the training. Any problems were noted, such as diagnoses missing in ICD-11 or skill-gaps to be addressed in future training. To avoid any delays to the patient discharge process, support team phone numbers were available at the workstations to provide around-the-clock support.

#### 2.2.3. Data Collection

Data were collected from the discharge summaries on the EHIS of all inpatients discharged from the internal medicine department between 1 April through 31 July 2021. Data were collected on:

Diagnosis-Related Variables

For each diagnosis, we collected the free text diagnosis, the ICD-11 entity, and the ICD-11 code. We also collected the body of the discharge summary for each case, which is documented in free text on the EHIS. The ICD-11 URI was recorded from 16 June onwards.

User Experience-Related Variables

A brief user experience survey to rate the ICD-11 experience for each discharge appeared in a pop-up once the physician attempted to save and exit the diagnosis screen. It included three mandatory multiple-choice questions: “Did you find what you were looking for?”, “How easy was it to find what you were looking for?”, and “Rate the time you took to find what you were looking for”. An additional optional free text box was provided for physicians wishing to add any more comments. The survey was offered for all discharges until 15 June 2021.

Other Variables

Data was collected on patient demographics, dates of admission and discharge, and discharge status. The physicians’ EHIS user ID, gender, and job position were also collected.

### 2.3. Data Analysis

We planned to report on the experience from three different perspectives: the data perspective looking into ICD-11 as a classification (by analyzing the diagnosis-related variables); the user perspective (by analyzing the user experience-related variables); the lessons learned for future implementation. We have dedicated this paper to reporting on the user perspective and the lessons learned; another publication is planned for reporting on the analysis of diagnosis-related variables.

For statistical analysis of the user-perspective variables, categorical data were described using number (n) and percentage (%). To determine whether a physician’s job position affected the odds of their ability to find the ICD-11 entity, the ease of finding it, and the time taken to find it, physicians were categorized according to their job position into two categories: junior (assistant registrars) and senior physicians (registrars, senior registrars, and specialists). This was followed by univariate ordinal logistic regressions where assistant registrars were the reference category. The associated effect was quantified using proportional odds ratios (pOR) and 95% confidence intervals (95% CI). As physicians discharged multiple patients (198 physicians answered the survey for 2424 discharges), in the ordinal logistic regression we used generalized estimating equations to adjust for the correlations among ratings from the same physician. Surveys for which the job position of the discharging physician was missing (n=62, representing 2.6% of discharges) were excluded from the regression analysis. Statistical analysis was performed using Statistical Package for the Social Sciences (IBM-SPSS version 22, Chicago, Il, USA).

## 3. Results

The pilot was implemented from 1 April to 31 July 2021. During this period, 3903 inpatients were discharged from the internal medicine department. A total of 241 physicians took part in the pilot.

### 3.1. The User-Experience Survey

From 1 April to 15 June 2021, the user-experience survey was completed for 2424 discharges by 198 discharging physicians. Of these, 1848 (76.2%) were discharged by an assistant registrar, 494 (20.4%) by a registrar, 19 (0.8%) by a senior registrar, and one discharge was completed by a specialist. The job title was missing for 62 discharges (2.6%). The number of surveys (discharges) per physician ranged from 1 to 49.

Overall, physicians reported that they were able to find the exact ICD-11 diagnosis they were looking for in 46.5% of discharges, partially in 37.1%, and not at all in 16.5%. The ease of finding the ICD-11 entity was perceived by physicians to be fairly easy in 47.1% of discharges, moderate in 36.3%, and difficult in 16.5%. The time taken to find the ICD-11 entity was considered by physicians as acceptable in 46.5% of discharges, “Ok” in 37.0%, and unacceptably long in 16.5% (Table 1).

Ordinal logistic regression, with generalized estimating equations to adjust for the correlations among ratings from the same physician, showed that job position had no significant effect on the odds of a physician’s perceived ability to find the ICD-11 entity (pOR = 0.99; 95% CI: 0.57, 1.72; *p* = 0.962), the ease of finding the ICD-11 entity (pOR = 1.10; 95% CI: 0.59, 2.06; *p* = 0.756), or the time taken to find the ICD-11 entity (pOR = 0.99; 95% CI: 0.57, 1.72; *p* = 0.965).

These results show that physicians viewed their ICD-11 experience favorably (around 83% overall positive response), and that this was comparable across all job positions.

Only 49 surveys (2.0%) were accompanied by a free text comment. Of these, 31 (63.3%) were positive comments on the new changes, 5 (10.2%) were negative comments (that the old system was better), 4 (8.2%) comments were unrelated to the pilot (IT issues or patient-related issues), and 9 (18.4%) comments related to difficulty in finding the ICD-11 entity. Three of the ICD-11 comments related to one diagnosis: cerebrovascular stroke (CVA), which prompted proposal submission on the ICD-11 maintenance platform. The other six comments reflected knowledge gaps that can be resolved through training on using the coding tool since not all physicians had attended the training.

### 3.2. ICD-11 Proposals

We submitted six proposals from the pilot experience. Each of these aimed to resolve a problem in the content of ICD-11 encountered when using version 05/2021 of ICD-11 for Mortality and Morbidity Statistics (ICD-11 MMS), which was the latest update of the ICD-11 blue browser at the time of the pilot [17]. Some were problems encountered by physicians as they documented diagnoses, and some were encountered as we prepared the hands-on exercises and training material for the pilot. All six proposals have been accepted and implemented on the ICD-11 maintenance platform (orange browser) [18] and are yet to make it to the next annual update of the official release of the ICD-11 MMS (blue browser). The implemented proposals are:

Proposal 1

Adding “haemorrhagic CVA NOS” as a synonym/index term under “Intracerebral haemorrhage”, which can now be found at “8B00.Z Intracerebral haemorrhage, site unspecified”.

Proposal 2

Adding “ischaemic CVA” as a synonym/index term under “Cerebral ischaemic stroke”, which can now be found at “8B11 Cerebral ischaemic stroke” (Foundation URI: http://id.who.int/icd/entity/636274910).

The rationale for proposals 1 and 2 is: Searching the coding tool for “CVA” yielded only “CVA not known if hemorrhagic or ischemic”, but the search results included neither “ischemic CVA” nor “haemorrhagic CVA”. That is because the abbreviation “CVA” was only included in the index terms under “Stroke not known if ischaemic or haemorrhagic” but not under “Cerebral ischaemic stroke” or “haemorrhagic stroke NOS”.

Proposal 3

Adding “gallstone with chronic cholecystitis” and “gallstone of gallbladder with chronic cholecystitis” as synonyms under “Cholelithiasis with chronic cholecystitis” (Foundation URI: http://id.who.int/icd/entity/1766545972), which is an index term under “DC11.1 Calculus of gallbladder or cystic duct with other cholecystitis”.

Rationale: Searching the coding tool for “acute cholecystitis with gallstones” gives results because gallstones, and calculus (both of which are synonymous) are included under that entity “DC11.0 Calculus of gallbladder or cystic duct with acute cholecystitis”. However, for the chronic alternative, “calculus of gallbladder with chronic cholecystitis”, only the term “calculus” is used. Since it is very common to use the term “gallstones” in the chronic case, searching for “chronic cholecystitis with gallstones” yielded no search results.

Proposal 4 to 6

Three proposals related to adding the term “cancelled” as a synonym of “not carried out” under the three entities:

- “QC11 Procedure not carried out due to patient’s decision for reasons of belief or group pressure” (Foundation URI: http://id.who.int/icd/entity/)
- “QC12 Procedure not carried out because of patient’s decision for reasons other than belief or group pressure” (Foundation URI: http://id.who.int/icd/entity/912390512)
- “QC10 Procedure not carried out because of contraindication” (Foundation URI: http://id.who.int/icd/entity/663184411)

Rationale: Many physicians commonly use the term “cancelled” rather than “not carried out” and this synonym was already available in ICD-10. Searching for “cancelled procedure” produced no search results.

### 3.3. Lessons Learned and Other Considerations

The lessons learned in terms of facilitators, challenges and other considerations are summarized in Table 2 and Table 3, and will be detailed in the discussion section.

## 4. Discussion

This pilot showed that ICD-11 can be successfully implemented for recording discharge diagnoses by physicians using the coding tool installed on the EHIS, while the codes and URIs are saved into the database. Overall, physicians were satisfied with the ICD-11 experience, irrespective of their job position. More than 83% positive response was reported for all three questions in the user-experience survey (the ability to find ICD-11 entity, as well as the ease, and time taken to find it). The pilot also showed that using ICD-11 in real-life is an effective way to improve the content of ICD-11 as a disease classification through feedback from users all over the world.

Implementing ICD-10, which was based on using books (the Alphabetical Index followed by the Tabular List) by medical coders in the medical records department, is a totally different experience from implementing ICD-11 by installing the ICD-11 API on EHIS to be used by physicians for documenting diagnoses. Since this is a novel approach for using ICD, we present the lessons learned during this pilot in terms of facilitators, challenges, and other considerations from the Kuwait public hospital perspective.

### 4.1. Facilitators

#### 4.1.1. National Health System Influence

The national health data automation project in Kuwait was the driving force for initiating the transition to ICD-11. Transitioning from a paper-based system for managing health data to an automated system that collects data from electronic health information systems and integrates databases from different health services necessitated the use of a disease classification system that is digital health-compatible. ICD-11 is best suited for this purpose because it can be integrated into local EHIS irrespective of the software used and provides semantic interoperability [1].

One of the long-term plans for the health system in Kuwait is to have a casemix/DRG system, as the country considers different options for a health financing model. Casemix systems, also known as DRGs (Diagnosis-Related Groups), are systems used to group patients into clinically meaningful groups (based on ICD codes) which are cost-homogenous. They are used for budget allocation, payment, benchmarking, and performance measurement. Each country uses a different DRG system based on the needs of its health system [19]. Most DRG systems use country-specific modifications of ICD-10, such as ICD-10-AM (the Australian modification) or ICD-10-CM (the American modification), which were developed to address country-specific needs by adding details lacking in WHO ICD-10. Adopting a DRG system that uses a country-specific modification poses a threat to the comparability of clinical morbidity data and makes the system vendor locked-in. ICD-11 avoids this problem, as it acts as a meta-database that incorporates all country-specific modifications under one unified classification system [4]. As such, ICD-11 includes a level of detail that enables it to be potentially used for a DRG system [1]. Kuwait has always used unmodified freely-available WHO ICD-10 for statistical purposes for both mortality and morbidity and is therefore free to make the move to ICD-11 without any impact on current health system financing or incurring extra costs.

In the absence of a DRG system in Kuwait, clinical coding for morbidity data was traditionally used for statistical purposes, not for reimbursement. Therefore, coding is not considered a desirable career path for most HIM graduates. This has resulted in a lack of qualified coders in MOH and the absence of “medical coder” as an official job title. However, with the ongoing automation project and future plans to have a DRG system, the quality of clinical coding is becoming more important. Transitioning to ICD-11 does not necessitate investment in recruiting/training the number of coders needed for coding more than 230,000 inpatient discharges, three million outpatient visits, and 20 million primary healthcare visits per year [8]. Instead, as the physician uses the ICD-11 coding tool to document the diagnosis using their own preferred terminology, the ICD-11 code is automatically saved (with the URI) into the database [1]. When the resulting ICD codes are used for the purpose of statistical reporting, this method is likely to improve the quality of data, but when needed for DRGs, coding rules must be applied. However, even in the second scenario, fewer coders would be required, as their role would focus on auditing and applying coding rules and not on converting the diagnoses into ICD codes.

#### 4.1.2. Leadership Commitment and Support

Leadership commitment and support from the director of NCHI, the head of WHO-FIC CC, the hospital director, the head of the internal medicine department, the head of central IT department at MOH, and the WHO ICD-11 team were crucial facilitators. ICD-11 implementation requires prior ICD-11 training for those who will implement the pilot, training to install the ICD-11 API onto the EHIS, IT resources (computers, internet), dedicated on-site support staff, and the involvement of physicians—all of which require formal approvals and resources. Furthermore, leadership support facilitated the collaboration and communication between different departments (NCHI, IT, and internal medicine). This corroborates findings from other studies involving interdepartmental collaboration in healthcare projects. A study in 47 USA-based hospitals also listed leadership support as the number one facilitator of successful interdepartmental quality improvement [20].

To guarantee leadership support, we held an initial meeting for all stakeholders to highlight the benefits of implementing ICD-11 and its potential use cases. The messages delivered to each stakeholder to persuade them of the change were tailored around answering one question from their perspective: “what’s in it for me?”. Evidence from scientific literature on change management proves that tailoring messages to fulfil the needs of different types of audience improves their engagement [21,22].

The support of the WHO included ICD-11 ToT training to NCHI staff, a webinar on the technical infrastructure of ICD-11 for the IT team, several virtual meetings to discuss the pilot plan with NCHI staff, and organizing virtual meetings with countries in the Eastern Mediterranean region who have implemented ICD-11 pilots so that they could share their experiences, such as in the case of the primary healthcare pilot by the United Nations Relief and Works Agency (UNRWA) for Palestine Refugees in the Near East [23].

#### 4.1.3. Team Approach

To ensure smooth implementation, a multidisciplinary team was formed to plan the pilot. The team included representatives from all stakeholders: NCHI, hospital administration, hospital IT, and a physician from the internal medicine department. Involving physicians from the hospital who are familiar with the local work environment and culture and who would be a part of the change, guaranteed the acceptance of the change by their peers. This corroborates findings from a Swedish study on responses to change in healthcare, which concluded that healthcare professionals tended to be more involved in or support changes which they initiated themselves or featured their active input [24].

#### 4.1.4. Training and On-Site Support

The evolution of ICD coding from book-based ICD-10 to the completely electronic ICD-11 should be accompanied by a similar evolution in training methods, such as the use of social media platforms. Moreover, the target trainees in ICD-11 (physicians) work in a different environment from those of ICD-10 (medical coders), dictating a different approach to training in terms of the training content, duration, medium, and dissemination.

Training physicians in non-clinical skills, such as using ICD-11 for documenting diagnoses, is a challenging task due to their busy schedules. The NCHI team kept the in-house training sessions short (single two-hour workshops). Furthermore, because ICD-11 training is based on displaying how to use the ICD-11 coding tool on a computer screen, videos are a convenient medium for illustrating these steps. We recorded two ten-minute videos based on the training material. The training videos were then distributed on the social media platform commonly used by physicians to communicate in the hospital, which was WhatsApp. A widely available smartphone application, WhatsApp is ideal for modern medical education [25]. It enabled us to reach a large number of physicians in a short period of time, with minimal disruption to their work schedule. This was especially helpful due to COVID-19 social distancing restrictions and physicians already overstretched with the burden of the pandemic. WhatsApp has previously been used as a means for physician medical education in other training programs, such as that published by the Duke University Cardiovascular Education Group [26].

To reach an even wider audience and to share the training material with others planning their ICD-11 pilots, NCHI created a YouTube channel and uploaded the training videos in addition to other training material on ICD-11 [16]. A recently published literature review also highlighted the need for health care institutions to employ the potential of social media platforms, including WhatsApp and YouTube, for medical education [25].

To reduce potential resistance to change, the training was especially tailored for hospital physicians who are generally keen on understanding the changes made to their own EHIS (the new diagnosis screen that they will have to use) more than anything else. That is why we used screenshots from the hospital EHIS for the training material to induce a sense of familiarity and to focus the concentration of the trainees (physicians) on the most important change, namely ICD-11. ICD-11 was mainly presented as a standard way for documenting diagnoses, not as a coding system. The use of coded data for research, resource allocation, and decision making was mentioned as an outcome of documenting diagnoses using ICD-11, but this was not the main focus. The user-friendly ICD-11 features most appealing to clinicians, such as abbreviations, synonyms, and the availability of up-to-date clinical diagnoses, were all underscored during the training to gain physician interest and buy-in. To encourage physicians to attend the training, we awarded attendees with Continuous Medical Education (CME) credit points. An accumulation of a certain number of CMEs is required by MOH for physicians’ promotion and/or issuance/renewal of license to practice.

On-site support was available for physicians to quickly resolve any ICD-11 or IT issues that may arise. This helped improve acceptance of ICD-11 among physicians by avoiding delays in discharging patients and provided physicians with the opportunity of one-to-one training by NCHI staff. During our daily rounds, we often observed Peer Assisted Learning (PAL) at the computer workstations, with physicians who had attended the training aiding their peers who had not. This more-sustainable form of training was encouraged, as it ensures continuous education even when NCHI staff are not available. It also helps to build confidence in physicians when using the ICD-11 coding tool since their peers appear confident enough to share their new gained skills in ways that are easy for their colleagues to understand [27].

#### 4.1.5. Other Changes in the Final Diagnosis Screen

ICD-11 is a tool for documenting diagnoses in a standardized way while automatically saving the ICD code in the database. However, ICD-11 codes will only truly reflect the final diagnoses at discharge if the correct diagnosis is documented by the physician. Implementing ICD-11 onto the system was a chance for NCHI to improve the final diagnosis screen by implementing a simple change, namely distinguishing between the principal and the other (secondary) diagnoses. This was also accompanied by dedicating one of the two training videos to the definitions and the most basic guidelines for documenting final inpatient diagnoses. Due to the lack of a DRG system, Clinical Documentation Integrity (CDI) is not part of the physicians’ culture. This minor change could form the beginning of future training in CDI to improve the quality of documentation even before a DRG system is implemented. Other ICD-11 implementation projects, such as that by the UNRWA, also found a need to make changes to their electronic medical record screens when implementing ICD-11 [23].

### 4.2. Challenges and Other Considerations

#### 4.2.1. Procedure Coding and DRG Systems

Although the possibility exists for building a DRG system based on ICD-11, this needs a supporting procedure classification. The International Classification of Health Interventions (ICHI) is the freely available WHO tool for reporting health interventions across health services, including diagnostic, medical and surgical interventions. Like ICD-11, ICHI uses combinations of stem codes and extension codes to add granularity and can form packages of interventions performed together. In October 2020, the Beta-3 version of ICHI was released, and although the component relating to clinical interventions has been finalized, an officially released version is yet to be issued by the WHO [5,28]. A work-in-progress version can be accessed on the WHO-FIC maintenance platform (orange browser) [29]. Some countries have already started using ICHI to code procedures. Egypt adopted ICHI as the coding system used in service packages for its Universal Health Insurance (UHI) program. They created lists of essential interventions in all specialties to be transformed to the electronic information systems at different health facilities [30]. However, using static lists misses out on the advantages of having a dynamic API installed on the EHIS, which is the whole point of the latest WHO-FIC which makes them suitable for today’s digital era. The imminent development of an ICHI API will open the possibility for its installation on EHIS, so that it can be used in a similar way to ICD-11 [31].

In countries with established DRG systems, ICD and procedure codes have financial importance, as they are the basis for provider reimbursement. While these countries may find transitioning to a new system challenging, in Kuwait, we have a more liberal approach since we do not yet have a DRG system. A freely available and health system-customizable DRG system based on ICD-11 and ICHI codes is still to be developed with the help of the WHO.

#### 4.2.2. Physicians as ICD-11 Users

Short training sessions were more convenient for physicians’ busy schedules, but did not provide enough time to delve into chapter-specific details. Moreover, physicians are only interested in using ICD-11 as a means for standardizing diagnosis documentation to replace free text, not in applying the guidelines for morbidity coding and reporting, which would be of paramount importance if a DRG system was implemented. It is therefore essential, in such case, to have a team of experienced medical coders who are familiar with those guidelines to ensure that the principal diagnosis was correctly assigned and that all relevant secondary diagnoses were documented. The benefit of physicians using ICD-11 is that it requires fewer medical coders, as the code for each diagnosis is assigned automatically when the physician uses the ICD-11 coding tool to document the diagnosis. This means a shift in HIM professionals’ time and skills towards much needed roles, such as auditing and CDI.

#### 4.2.3. Quality of Clinical Documentation

ICD-11 has the potential to improve the quality of diagnosis documentation by providing diagnosis-specific postcoordination options. Comparing results from different morbidity pilots must be done with caution, as the prior quality of documentation by physicians (before ICD-11 implementation) should be taken into consideration. As such, results from ICD-11 morbidity pilots will not only reflect ICD-11 as a classification but will also be confounded by the prior quality of clinical documentation. When using ICD-11, physicians in countries where ICD codes are already used for billing are more likely to use postcoordination to add details to their diagnoses relative to countries where ICD codes are only used for statistical reporting.

#### 4.2.4. IT Issues

Since ICD-11 is fully electronic, it depends on information technology (IT) infrastructure. IT issues are unrelated to ICD-11 as a classification but are important for its implementation. It is necessary that IT problems are promptly resolved, otherwise we risk physician dissatisfaction with the whole experience, as physicians may come to associate ICD-11 with EHIS problems. We encountered IT problems during the small-scale pilot, and we only started the actual pilot when IT issues were settled.

The hospital uses a web-enabled EHIS. A Docker container was used to deploy the ICD-11 API on the EHIS and Google Chrome browser was launched in the container to access the coding tool. The coding tool is not directly embedded into the discharge screen; instead, the tool is accessed on a second window that opens when an icon beside the ICD-11 field on the discharge screen is clicked. This means that unnecessary extra steps are required: retyping the diagnosis into the search box on the new window (or copying and pasting the free text diagnosis from the diagnosis screen before opening this window), after clicking the selected ICD-11 entity, the physician must also click save, and then another click is needed to close this window. Directly embedding the ICD-11 API into the ICD-11 field on the diagnosis screen would have made the process seamless from the users’ perspective by reducing unnecessary steps and clicks.

### 4.3. Strengths of the Study

This is the first paper reporting on real-life ICD-11 implementation using the coding tool embedded into the EHIS for documenting inpatient discharge diagnoses by physicians in a public general hospital. We piloted the whole process of ICD-11 implementation, including engaging stakeholders, preparing the IT infrastructure, training physicians on ICD-11, and real-time use of the ICD-11 coding tool for documenting diagnoses. With 241 physicians and more than 3900 inpatient records over a period of four months, this pilot reflects the practical aspects of using ICD-11 for morbidity in a dynamic environment, showing how it can become part of the routine documentation of discharge diagnoses on the EHIS. We have shared the lessons learned from this pilot, highlighting facilitators and challenges, and captured the voice of physicians interacting with ICD-11.

This pilot has also been a learning experience for the NCHI team, which also functions as a WHO-FIC CC. Creating a YouTube channel allowed us to share our gained knowledge and skills not only with the pilot participants but also with a wider audience. Educational videos on topics such as how to submit ICD-11 proposals and an introduction to ICD-11 coding are freely accessibly on the channel [16]. A short video on the channel provides instructions to anyone interested in trying out an ICD-11 exercise on how to join the Kuwait center on the ICD Field Implementation Tool (ICD-FIT) platform, which is a WHO web-based application that can be used by trainees to solve exercises on ICD-11 and receive feedback on their answers. Another way of sharing this experience was presenting it in WHO-organized meetings on ICD-11 both regionally (GCC and Eastern Mediterranean) and internationally (WHO-FIC Annual Network Meeting).

### 4.4. Limitations

Unlike mortality coding, which is reported to the WHO using internationally standardized rules and guidelines, morbidity coding is mainly used at the national level. The use of morbidity codes, as well as the rules and guidelines for coding and reporting vary, widely between different countries based on the requirements of their health systems [1]. Our results report on the ICD-11 pilot from the point of view of the health system in Kuwait, so the lessons learned may not all be relevant to countries with different health systems.

The pilot was only conducted in the internal medicine department, so the captured diagnoses and user-survey may not reflect other specialties. This paper does not report on the analysis of diagnosis-related variables collected during the pilot; this is planned for a future publication. Furthermore, it is important to note that the results of the user-survey relating to finding ICD-11 entities and the time taken to find them are based on the perceptions of physicians without objective verification by an independent reviewer. For example, where a physician says they were able to find exactly what they were looking for, we have not verified in this paper whether the ICD-11 entity they selected did indeed exactly match the actual diagnosis. It was not possible to identify how many physicians accessed the training videos shared on the WhatsApp chat groups or took part in PAL, and so we could not relate those numbers with the findings from the survey. We did not study physicians’ experience regarding the provided training, IT support, or on-site ICD-11 support.

### 4.5. Pilot Implications and Future Plans

The positive feedback from the pilot in the internal medicine inpatient wards provided by the physicians and head of department encouraged other department heads to request ICD-11 implementation in their departments. The hospital director was also supportive of scaling up implementation to cover diagnosis documentation for morbidity outpatients and inpatients in all hospital departments. The internal medicine department has not stopped using ICD-11 since the end of the pilot, making it the routine way for documenting diagnoses. To prepare for scaling up, eight ICD-11 in-house training workshops were conducted in October and November 2021 and an extra video on basic outpatient diagnosis documentation guidelines was distributed on WhatsApp. However, scaling up comes with its challenges, including the difficulty in using the hospital’s relatively small computer lab to cover such a large number of doctors. Virtual simulation was used to provide physicians the chance to do a practical hands-on exercise on the EHIS at their own convenience. Full implementation in the whole hospital started in December 2021.

Data on diagnoses would then be available in real-time from the hospital EHIS. Previously, the paper forms used in the medical records department were coded with ICD-10, then delivered to NCHI after the end of the month (commonly later). This was followed by data entry by clerks at NCHI, who were overwhelmed with the forms from all the other hospitals as well. Only when ICD codes are in the NCHI database and data quality checks are completed, can they be used for statistical reporting and research purposes.

ICD-11 training and implementation introduced physicians to the concept of distinguishing between the principal diagnosis and other diagnoses and the fact that there are rules and guidelines for selecting the principal diagnosis and what diagnoses should or should not be documented as other/secondary diagnoses for an inpatient episode of care. This opens up future prospects for developing specialty-specific guidelines to improve the quality of clinical documentation with a chance to focus in the future on what individual ICD-11 chapters can offer different specialties.

The data collected during this pilot included diagnosis-related variables, which will be analyzed and reported in another planned publication. Furthermore, different clinical departments in the hospital have already started discussing research on certain diagnoses now that diagnosis documentation is standardized and the data is electronically available and easily retrievable from the hospital EHIS.

The future plan is to start the national roll out of ICD-11. The first step towards that goal would be to showcase the Farwaniya hospital example, as a hospital that has fully adopted ICD-11 for morbidity, to other hospitals in the country in a meeting with the MOH undersecretary, other hospital directors, and NCHI. Kuwait has seven MOH general hospitals and 14 tertiary hospitals [8]. Getting leaders to listen to the experiences of the director of the hospital himself and the heads of departments is expected to encourage implementation in other hospitals.

## 5. Conclusions

ICD-11 can be successfully implemented for documenting discharge diagnoses by physicians in inpatient wards in a public hospital on the EHIS using the coding tool, while ICD-11 codes and URIs are automatically recorded in the database.

Piloting ICD-11 for morbidity ideally proceeds via the following steps: determining the Center of Excellence, engaging stakeholders, selecting the setting, building a common understanding of the discharge process in the selected setting, evaluating and preparing IT infrastructure, ICD-11 training, pre-pilot testing on a small scale, and implementing the pilot while providing on-site support and collecting data for analysis.

Changing the ICD coding process from medical coders using ICD-10 to physicians using ICD-11 is an organizational change requiring an effective communication strategy that typically follows three phases [21]. The first phase, readiness, involves convincing leaders to support the change using stakeholder-tailored messages. In adoption, the second phase, physicians experiment with using ICD-11 in real-life while voicing their opinions using a simple structured feedback mechanism. Training for this phase must be customized to physicians’ needs, interests, and language and disseminated using a familiar medium. The final phase, institutionalization, is ultimately reached when the change is accepted and maintained, with the success of the adoption phase being communicated to other hospital departments to scale up implementation, making ICD-11 the routine way for documenting and reporting diagnoses.

## Figures and Tables

**Table 1:** Results of user-experience survey (N = 2424).

	Frequency	Percentage
Did you find what you were looking for?		
Yes, exactly	1126	46.5
Yes, partially	899	37.1
No	399	16.5
How easy was it to find the diagnosis in this case?		
Fairly easy	1142	47.1
Moderate	881	36.3
Difficult	401	16.5
The time you took to find what you were looking for was		
Acceptable	1126	46.5
Ok	898	37.0
Unacceptably long	400	16.5

**Table 2:** Facilitators.

Facilitators	
National health system influence	National automation project (transferring from paper-based system for national reporting to an electronic one) National health system requirements: need for a DRG system using WHO classifications National challenge: lack of experienced qualified medical coders
Leadership commitment and support	Tailor stakeholder-specific messages for persuasion Benefit from leadership commitment to guarantee formal approvals, resources, and effective communication between different stakeholders
Team approach	Multidisciplinary team involving representatives of all stakeholders Users (physicians) accept change when involved in making decisions
Training	Short duration Uses screenshots from own hospital EHIS Variable options to access (attend in person, watch video) Wide dissemination using social media (WhatsApp, YouTube) Peer Assisted Learning (PAL) Speaks the language of physicians
On-site support	ICD-11 support IT support
Other changes in the final diagnosis screen	Diagnosis screen improvements to maximize benefit from implementing ICD-11

**Table 3:** Challenges and other considerations.

Challenges and Other Considerations	
Procedure coding and DRG systems	Only Beta version of ICHI available No ICHI API yet Awaiting DRG system that uses both ICD-11 and ICHI
Physicians as ICD-11 users	Lack of interest and insufficient time for training on guidelines for morbidity coding In case of DRG system, a number of experienced qualified coders are still required
Prior quality of clinical documentation	Not comparable across different countries Affects physicians use of coding tool features (e.g., postcoordination)
IT issues (problems)	Impact physician satisfaction with ICD-11

## Data Availability

The dataset used and analyzed for this study, which includes patient and physician information, is owned by the Ministry of Health, Kuwait. The authors do not have the right to share the data. Permission to use the data by the authors was officially granted by the Standing Committee for Coordination of Health and Medical Research, Ministry of Health (no. 1888/2021). Interested researchers can gain access to the data upon reasonable request after obtaining required permits from the committee. Contact information: email: ethics@moh.gov.kw or PO Box (5) 13001, Safat, Kuwait. Tel.: 0096524622228–0096524622230, Fax: 0096524866514. Address any requests specifically to the “Head of the Standing Committee for Coordination of Health and Medical Research”.
